# Brain structure correlates of expected social threat and reward

**DOI:** 10.1038/s41598-020-74334-z

**Published:** 2020-10-22

**Authors:** Bonni Crawford, Nils Muhlert, Geoff MacDonald, Andrew D. Lawrence

**Affiliations:** 1 Cardiff University Brain Research Imaging Centre (CUBRIC), School of Psychology, Cardiff University, Maindy Road, Cardiff, CF24 4HQ UK; 2 Division of Neuroscience and Experimental Psychology, University of Manchester, Manchester, UK; 3 Department of Psychology, University of Toronto, Toronto, Ontario Canada

**Keywords:** Reward, Cognitive control, Motivation, Perception, Personality, Risk factors, Human behaviour

## Abstract

Prospection (mentally simulating future events) generates emotionally-charged mental images that guide social decision-making. Positive and negative social expectancies—imagining new social interactions to be rewarding versus threatening—are core components of social approach and avoidance motivation, respectively. Interindividual differences in such positive and negative future-related cognitions may be underpinned by distinct neuroanatomical substrates. Here, we asked 100 healthy adults to vividly imagine themselves in a novel self-relevant event that was ambiguous with regards to possible social acceptance or rejection. During this task we measured participants’ expectancies for social reward (anticipated feelings of social connection) or threat (anticipated feelings of rejection). On a separate day they underwent structural MRI; voxel-based morphometry was used to explore the relation between social reward and threat expectancies and regional grey matter volumes (rGMV). Increased rGMV in key default-network regions involved in prospection, socio-emotional cognition, and subjective valuation, including ventromedial prefrontal cortex, correlated with both higher social reward and lower social threat expectancies. In contrast, social threat expectancies uniquely correlated with rGMV of regions involved in social attention (posterior superior temporal sulcus, pSTS) and interoception (somatosensory cortex). These findings provide novel insight into the neurobiology of future-oriented cognitive-affective processes critical to adaptive social functioning.

## Introduction

Making friends—and/or forming romantic partnerships—is of critical importance for adults' adjustment to new environments, for instance, starting university^1^. Humans are therefore intrinsically motivated to actively seek out and affiliate with others, with the aim of fostering new social connections^2^. By their nature, however, social interactions with unfamiliar others simultaneously offer the prospect of both rewards (e.g. having a pleasant conversation, feeling a sense of belonging)^2^ and threats (e.g., feeling embarrassed, being socially rejected)^3,4^.

Neurobehavioral motivation frameworks posit two basic systems that mediate actions geared towards desirable and undesirable outcomes—an approach (or behavioural activation) system (BAS) and an avoidance (or behavioural inhibition) system (BIS), respectively^5–7^. These are suggested to be independent neurobehavioral systems, which may compete to drive behaviour^5,8^. Models of social motivation connect these basic approach/avoidance motivational processes with social cognition, including attentional focus and beliefs about other people's behaviour in social interactions^4,9,10^.

People differ in their sensitivity to social reward and threat and such inter-individual differences are relatively stable^11^, although such sensitivities be heightened during adolescence^12,13^. These stable traits are associated with the likelihood of being socially connected or, conversely, isolated^4,14^. It seems plausible that individual differences in these neurocognitive systems might exist on continua of shyness and sociability, respectively, with the extreme ends of these continua being clinically relevant^15,16^.

An emerging literature details neural responses to rejection or connection experiences and visual cues of social reward or threat^4,17–19^. An abundance of research implicates the amygdala in threat processing, including social threat^20^, and notably increased amygdala volume has been linked to behavioural inhibition and social anxiety^21,22^. The amygdala works in concert with pSTS in mediating sustained vigilance for signs of social threat^23^. The pSTS and amygdala are also active when recalling and reliving social evaluative threat situations^24^. By contrast, vmPFC is considered a “hub” of reward processing, including social reward^17,25^ and interacts with core social cognition regions including dorsomedial PFC (dmPFC) to mediate the experienced and remembered reward (pleasure) of social belonging or approval^4,17^. vmPFC-amygdala functional connectivity is also implicated in the cognitive regulation of negative affect, including in the face of social rejection^26,27^. Previous work has found that greater wellbeing and more successful emotion regulation are associated with greater rGMV in vmPFC^28,29^.

There is reason, however, to think that *prospective* cognitive-affect representations are at the heart of these putatively distinct social reward and threat motivational systems. BAS or BIS have been theorized to be primarily future-oriented (e.g., mediating hopes and fears about future desirable or undesirable outcomes^7^). MacLeod^30,31^ argued that affect is directly related to cognition and that positive and negative future-related cognitions may best be perceived as two separate dimensions of experience. Such future-oriented emotion systems depend on the capacity for “mental time travel” (MTT) inherent in episodic memory^32,33^. MTT enables vivid, detail-rich simulations of future events based on the flexible re-combination of episodic memories and newly generated images constructed by drawing on episodic memories combined with components of semantic memory, such as beliefs and schemas. Through the vivid imagination of future events, humans generate embodied predictions of events’ emotional impacts before their occurrence, which act as powerful motivators of behaviour^33,34^.

The capacity for MTT is mediated by a ‘core’ network, largely overlapping with the default-network, notably including vmPFC as a core node^32,35^. vmPFC is implicated, alongside medial temporal lobe regions, both in the construction of episodic memories and imagined future events (in part by accessing relevant schematic knowledge), as well as in their affective valuation based on current needs and goals^25,32,35^.

While recent research has studied individual differences in anticipated social reward and threat separately (e.g.^19,36,37^.), to our knowledge, no neuroimaging research has directly examined *both* individual differences in future-oriented social reward and threat expectancies in the context of fostering new social connections. Building on work in the domain of close relationships^9,38,39^ we developed a new instrument to examine inter-individual differences in reward and threat expectancies in the context of an imagined self-relevant social interaction with unfamiliar peers. This novel measure, the levels of dispositional expectancies for social threat and reward scale (LODESTARS), asks participants to vividly imagine that they have joined a new group, club, or society, then make predictions about the probable emotional consequences of these novel interactions and report their anticipatory and anticipated cognitions and emotions.

Individuals’ social reward and threat expectancies as measured by the LODESTARS are stable over time^40^, are associated with other stable affective traits such as self-esteem, and may be grounded in temperament and attachment experiences^41^. Given this trait-like stability, we predicted that individual differences in expectancies for social threat and reward would be associated with stable, structural aspects of the brain. Recent structural magnetic resonance imaging (sMRI) studies indicate that several aspects of real-world social behaviour are reflected in brain macrostructure (regional grey matter volume, rGMV) as assessed by voxel-based morphometry (VBM)^42^.

Here, we used VBM and an unbiased, whole-brain analysis to investigate the unique and overlapping rGMV correlates of inter-individual differences in social threat and reward expectancies (STE and SRE, respectively) using a combination of raw LODESTARS and LODESTARS scores that were orthogonalised (residualised) with respect to one another (see Methods for details). Further, to facilitate interpretation of the results, we examined (a) functional associations of rGMV peak voxels revealed by our analysis using the large-scale meta-analytic platform Neurosynth (https://neurosynth.org/) and (for key regions) (b) their structural covariance, which may reflect the extent to which brain regions belong to the same (or antagonistic) functional system(s)^43^. This was primarily an exploratory study. However, we made two tentative predictions, based on the close alignment of regional brain macrostructure and function^43^. First, given vmPFC involvement in the construction and valuation of events, including imagined personal future events, as well the more general finding that vmPFC activity scales with experienced and anticipated positive value^44^, we expected that vmPFC rGMV would correlate positively with SRE. In addition, given vmPFC involvement in emotion regulation, we also expected increased vmPFC volume to relate to lower STE.

Secondly, given their involvement in the processing of social threat and links to anxiety, we predicted that rGMV of the amygdala and posterior superior temporal sulcus (pSTS) would be positively correlated with STE.

## Methods

This study was approved by the Cardiff University Psychology Research Ethics Committee and was carried out in accordance with the relevant guidelines and regulations. All participants provided written informed consent in accordance with the Declaration of Helsinki.

### Participants and procedure

A power analysis^45^ indicated a sample size of n = 82 was required to detect a medium sized correlation (r = 0.3, alpha = 0.05, power = 0.8). One hundred right-handed healthy volunteers participated (74 female, 26 male, mean age 24 years, range 18–54). Participants completed a battery of measures including the LODESTARS, administered using Qualtrics (Provo, UT, https://www.qualtrics.com). Participants attended the imaging centre on a separate occasion for MRI.

### Measuring dispositional social expectancies: The LODESTARS

The LODESTARS is a 10-item inventory examining the extent to which respondents expect to experience social reward and threat during an imminent vividly imagined social encounter with a group of unfamiliar peers. Participants are asked to imagine that they have joined a new group, club or society and that this evening they will be going to a social event organized by this group/club/society. Participants imagine that this will be the first time they will meet other people who are in the group/club/society. After noting down the name of the group/club/society they have chosen, participants indicate their anticipated and anticipatory cognitions and emotions about the upcoming imagined event, by responding to 5 threat items and 5 reward items on a 5-point Likert scale. These items include “*I will probably meet one or more people who I will like a lot*” and “*I am a bit worried about feeling embarrassed during these interactions*” (see https://osf.io/hq5sg/ for the full measure). Approaching unfamiliar others and establishing initial social connections are core tasks when transitioning into novel social environments (e.g. entering university), and a prerequisite for integrating new people into one’s social network^1,11^.

Expectancies about social interactions are partly situation-specific^46^; however, there is a component of them that is influenced by individuals' temperament and stable working models (schemas) of self and others^47^. The LODESTARS was designed to tap the stable component, by probing participants’ expectancies for interactions with peers (with whom the participant is motivated to interact) in a generic social event context. The scenario described in the LODESTARS is nuanced (it simultaneously holds the possibility for social reward and threat), and thus in line with existing measures in which participants imagine themselves in an emotionally ambiguous (future) scenario^48^. These measures are sensitive to individual differences in affective style^30^. We used an imminent imaginary scenario, since short-term predictions enhance the tendency to rely on episodic emotional information, relative to personal semantic knowledge (beliefs etc.)^30,49,50^.

Data from more than 1300 participants demonstrate that the LODESTARS has a two-factor (reward, threat) structure and excellent psychometric properties, including high test–retest reliability and measurement invariance across gender^40^. The LODESTARS yields two scores for each participant: a social reward expectancy (SRE) score and a social threat expectancy (STE) score, both of which can range from 1 (low) to 5 (high). The LODESTARS has excellent construct validity and appears to be sensitive in distinguishing different social cognitive-affective processing styles. For example, attachment anxiety is associated with heightened STE, while avoidant attachment is associated with reduced social SRE^41^. Qualitative data from a community sample confirmed that people find the LODESTARS to be highly naturalistic^40^, consistent with findings that people devote considerable time in daily life to imagining and evaluating social encounters^51^.

### Image acquisition

T1-weighted anatomical images for each participant were acquired using a 3-T GE HDx MRI scanner at Cardiff University Brain Research Imaging Centre (CUBRIC). The 3-D T1-weighted whole-brain images were acquired using a fast-spoiled gradient echo sequence (FSPGR) with 1 × 1 × 1 mm voxel size and between 168 and 182 contiguous slices. Image acquisition parameters were as follows: repetition time (TR) = 7.8 ms echo time (TE) = 2.984 ms; inversion time = 450 ms; flip angle = 15°; data matrix = 256 × 192. These data were usually acquired within one week of the participant completing the LODESTARS (mode = 3 days).

### Image analysis

Voxel-based morphometry (VBM) was performed using SPM12 (Wellcome Trust Centre for Neuroimaging, https://www.fil.ion.ucl.ac.uk/spm/software/spm12) implemented in MATLAB v. R2012b (The MathWorks). First each participant’s structural image was segmented into grey matter (GM), white matter (WM) and cerebrospinal fluid (CSF) using the ‘unified segmentation’ set of algorithms in SPM12. The image segments of interest (the GM segments) were then normalised to MNI space using the diffeomorphic anatomical registration through exponentiated lie-algebra (DARTEL) registration method in SPM12^52^. The GM images were smoothed using a Gaussian kernel of 8 mm full width at half maximum. An 8 mm smoothing kernel is optimal for detecting morpho-metric differences in both large and small neural structures^53^.

### Statistical analysis 1: LODESTARS VBM

We examined correlations between regional grey matter volume (rGMV) and social reward expectancy and social threat expectancies from the LODESTARS. We accounted for the potentially confounding variables of age and gender^54^ by entering them into the general linear models as ‘regressors of no interest’. Participants’ overall brain volumes were also accounted for, by means of proportional scaling in SPM12^55^. A binary MNI brain mask (SPM8 brainmask.nii) was used to restrict the analysed volume to voxels within the brain.

#### Model specification

Inference as to whether regional rGMV significantly correlates with one or both regressors of interest requires that *both* LODESTARS-reward and threat scores be included within the same model^56^.

There is debate as to the extent to which reward and threat expectancies are independent, both in terms of underlying brain substrates and as they manifest in behaviour/self-report^5,7^. It is informative, therefore, to clarify the effects on rGMV that are uniquely attributable to each of these two regressors. Entering both into a GLM will automatically achieve this: an essential property of the GLM is that only the variability unique to each regressor drives the parameter estimate for it, so that each effect is adjusted for all others^57^. Only assessing the rGMV associations of variance that is unique to threat and to reward carries its own problems however. These are due to the fact that the standard process of GLM parameter estimation removes the effects of shared variability^57^. When two regressors are highly correlated, their shared variability is large and the unique component for each is correspondingly small. This results in a loss of statistical power. Further, in this case, it is interesting to explore not only the regional rGMV differences uniquely associated with social threat or reward expectancies, but also those present when the shared variance is included within the model.

The correlation between LODESTARS-STE and -SRE scores in the present study was − 0.36, *p* = 0.0002 (95% CI = − 0.56 to − 0.137), indicating significant shared variance between these two regressors. To explore the shared as well unique variance, while maintaining the same degrees of freedom across models, we used orthogonalised LODESTARS scores in combination with raw scores. Orthogonalised scores are the residuals that result from regressing STE on SRE scores and vice versa. By definition, these constitute the portions of each LODESTARS score that are not predicted by the other LODESTARS score.

**Figure 1:**
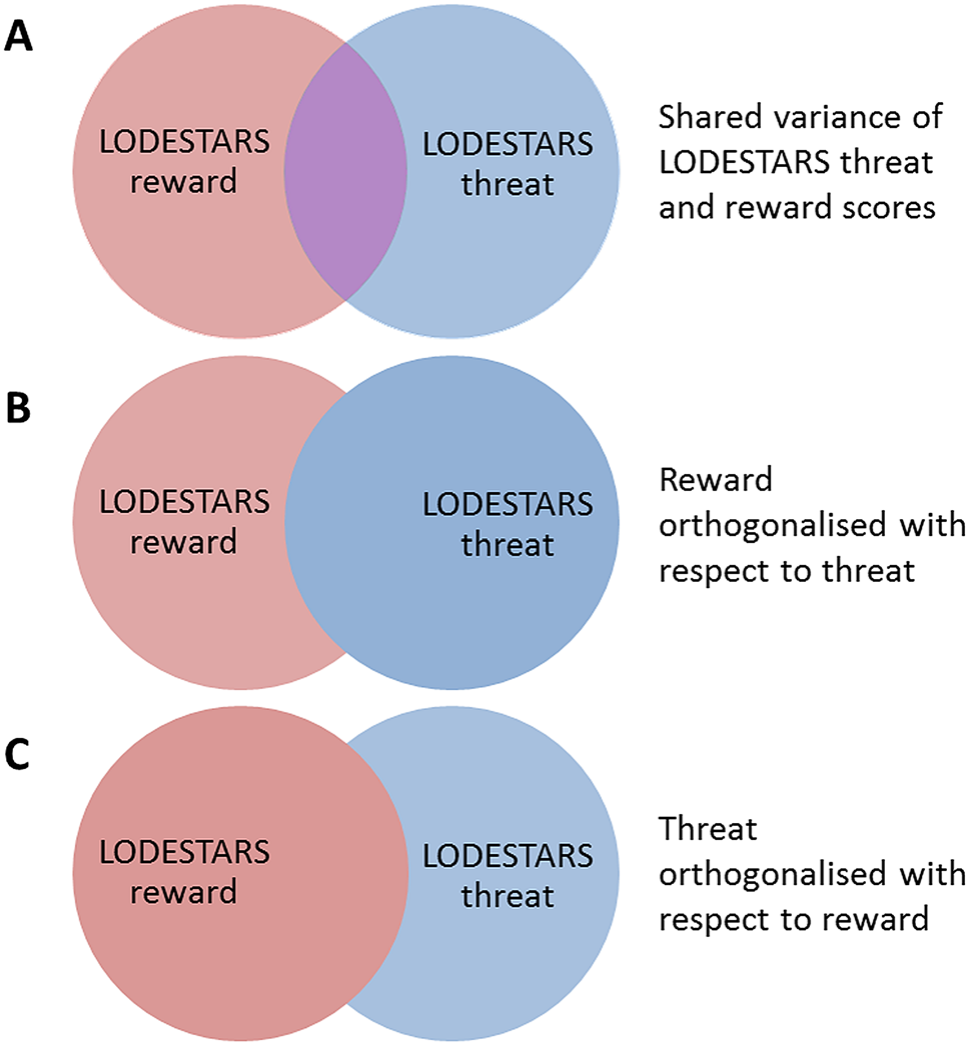
Venn diagrams illustrating how the variability is distributed across the 2 LODESTARS regressors where red is unique to SRE, blue is unique to STE and purple is shared. (**A**) depicts ‘raw’ LODESTARS-STE and SRE scores, which exhibit some overlapping variance. (**B**, **C**) depict the two regression models run, demonstrating the effects of variable orthogonalisation. In (**B)**, all the shared variance is assigned to LODESTARS-STE while in **C**, all shared variance is assigned to LODESTARS-SRE.

We conducted two GLMs, which between them allowed assessment of individual differences in rGMV uniquely attributable to variance in LODESTARS-STE or SRE, as well as rGMV associations present when the shared variance was included but attributed exclusively to social threat or reward. The two models are specified below. See Fig. 1 for a diagrammatic representation of the assignation of (shared) variance that results from orthogonalisation.

**Model** 1**: All shared variance assigned to social threat expectancies.**

$${\text{rGMV}} = \alpha + {\text{b}}_{0}\, {\text{LODESTARS}}\_{\text{reward}}\_{\text{orth}} + {\text{b}}_{{1}}\, {\text{LODESTARS}}\_{\text{threat}} + {\text{b}}_{{2}} \,{\text{age}} + {\text{b}}_{{3}} \,{\text{gender}}$$

**Model** 2**: All shared variance assigned to social reward expectancies.**

$${\text{rGMV}} = \alpha + {\text{b}}_{0}\, {\text{LODESTARS}}\_{\text{reward}} + {\text{b}}_{{1}} \,{\text{LODESTARS}}\_{\text{threat}}\_{\text{orth}} + {\text{b}}_{{2}}\, {\text{age}} + {\text{b}}_{{3}} \,{\text{gender}}$$

where LODESTARS_threat_orth = LODESTARS-STE orthogonalised with respect to SRE scores and LODESTARS_reward_orth = LODESTARS-SRE orthogonalised with respect to STE scores.

#### Correction for multiple comparisons

To correct for multiple comparisons across the whole brain, we applied non-stationary cluster extent correction as implemented in the VBM8 toolbox (https://dbm.neuro.uni-jena.de/vbm/) running in SPM12. We used 3DClustSim (AFNI) to calculate the overall expected voxels-per-cluster threshold for our data, for α = 0.05, *p* ≤ 0.001, based on the brain mask we used (SPM8 brainmask.nii). This gave an expected cluster size of ≥ 86 voxels.

#### Neurosynth meta-analytic decoding

We sought to gain insight into what aspects of functional paradigms are most frequently associated with the regions identified in our VBM analysis via functional decoding. Functional decoding is a quantitative, data-driven method by which researchers can infer which mental processes may be related to activation in a specific brain region (or set of brain regions) across published fMRI studies. We conducted meta-analytic decoding using Neurosynth, a platform for large-scale, automated synthesis of functional magnetic resonance imaging (fMRI) data (https://neurosynth.org), to find the functional terms most frequently associated with the peak voxels identified in the VBM analysis. To facilitate interpretation, the top 10 terms with the highest correlation values for each peak voxel were selected. Non-content terms (such as “MRI,” “statistical”) and anatomical terms (such as “prefrontal,” “MTL”) were excluded. Terms that were near-duplicates of terms already included in the list were removed, such as “emotional” and “emotions” if “emotion” was higher on the list. This left two or three functional terms for each peak voxel; these are reported in Tables 1 and 2.

**Table 1:** Clusters that survived nonstationary cluster extent correction: shared variance between threat and reward included.

LODESTARS variable	Direction of correlation	Anatomical region	Cluster size (voxels)	MNI coordinates			T-score	Reflects unique variance?	Neurosynth decoding results
				x	y	z			
Social reward expectancy	Positive	left dorsomedial PFC	171	− 1.5	48	48	4.03	No. Does not survive when shared variance allocated to threat	“imagine” “social”
Social reward expectancy	Negative	[no clusters survive threshold]	–		–		–	–	
Social threat expectancy	Positive	right posterior superior temporal sulcus (pSTS)	212	45	− 58.5	10.5	4.24	Yes. Survives when shared variance allocated to reward	“eye gaze” “action observation”
Social threat expectancy	Negative	right ventromedial PFC	282	3	69	− 4.5	4.01	No. Does not survive when shared variance allocated to reward	“Theory of mind” “resting state”
		left lateral inferior occipital lobe	90	− 28.5	– 90	− 6	3.80	Yes. Survives when shared variance allocated to reward	“face” “visual”
		right postcentral gyrus (somatosensory cortex)	187	60	− 12	30	3.58	No. Does not survive when shared variance allocated to threat	“somatosensory” “action observation”

**Table 2:** Overlap of clusters reflecting bipolar valence that survived *p* < 0.005, 86-voxel extent threshold.

Anatomical region	Extent of overlap (voxels)	MNI coordinates			T-value	Neurosynth decoding results
		x	y	z		
Right ventromedial prefrontal cortex	68	9	48	− 21	3.41	“social”, “reward”, “autobiographical”
Right lateral inferior temporal gyrus	90	61.5	− 13.5	− 36	3.39	“Theory of mind”, “social”
Right parahippocampal gyrus	19	27	− 25.5	− 31.5	3.04	“episodic”, “semantic”, “recollection”

### Statistical analysis 2: overlap analysis

To test for brain voxels in which rGMV is significantly correlated (positively or negatively) with social threat *and* reward expectancies, two further GLMs were applied. These models each contained only one LODESTARS variable as the regressor of interest. The same thresholding was applied as in statistical analysis 1: *p* < 0.005, with an 86-voxel cluster extent threshold.

These models yielded statistical parametric maps (SPMs) of brain regions in which rGMV correlated positively with SRE, positively with STE, and negatively with STE. (No clusters survive threshold for negative correlation with SRE). These gave rise to two overlap analyses: 1, {social reward-positive and social threat-negative} (henceforth ‘bipolar valence’) and 2, {social reward-positive and social threat-positive} (henceforth ‘salience’).

The combinations of SPMs were inspected for overlap by means of masking in SPM12.

### Statistical analysis 3: structural covariance analyses

Inter-individual differences in the macrostructure of a brain region often co-vary with inter-individual differences in macrostructure of anatomically connected regions—so-called structural covariance^43^. Like other forms of connectivity, inter-regional SC may reflect shared functional specialization^43^. Thus, to further characterize the network affinities of regions linked with SRE and STE, we examined potential grey matter SC between dmPFC and vmPFC, between vmPFC and amygdala, and between pSTS and amygdala.

We extracted GMVs for the peak voxels of the dmPFC, vmPFC and pSTS clusters that survived cluster-extent correction in the LODESTARS VBM. These voxels were used as seeds in the subsequent analysis.

Our target regions of interest (ROIs) were specified by masks: a bilateral amygdala mask created from the Caltech atlas of the human amygdala^58^ and a bilateral vmPFC mask created from the Neuromorphometrics atlas (Neuromorphometrics labels, Right and Left MFC medial frontal cortex)^59^. We used seed-based SC analyses^60^, conducted in SPM12, to identify voxels within our target ROIs in which GMV covaried with GMV in the seed voxel. Our analyses identified voxels in which target region GMV covaried positively with seed GMV, and (separately) voxels in which target region GMV covaried negatively with seed GMV. The effects of gender, age, and total brain volume were accounted for in these models. As this was a hypothesis-driven, rather than exploratory analysis, we employed more stringent correction for multiple comparisons than in analyses 1 and 2. Specifically, threshold-free cluster enhancement (TFCE), which controls the family-wise error rate at *p* < 0.05^61^.

## Results

The mean LODESTARS-SRE score in this sample was 3.7 (from a max. possible score of 5; range = 2.0–4.8); std. dev. = 0.49) and the mean LODESTARS-STE score was 3.3 (range = 1.0–5.0, std. dev. = 0.92). Cronbach’s alpha was 0.65 for LODESTARS-SRE and 0.87 for LODESTARS-STE. There were no significant gender differences in the LODESTARS scores. LODESTARS-SRE scores did not correlate with age, however LODESTARS-STE scores decreased with increasing age (*r* = − 0.30, *p* = 0.003, 95% CI = − 0.49 to − 0.103).

A paired-samples *t*-test indicated that the mean LODESTARS-SRE score was significantly higher than mean LODESTARS-STE score, *t* = 3.05, *p* = 0.003, d_av_ = 0.5.

### Statistical analysis 1: LODESTARS VBM results

First, correlations between rGMV and LODESTARS-STE/SRE were examined in the SPM T-maps in which shared variance was included. That is, the outputs of the STE orthogonalised with respect to SRE model were inspected for correlations between rGMV and LODESTARS-SRE scores. The outputs of the SRE orthogonalised with respect to STE model were inspected for correlations between rGMV and LODESTARS-STE scores. Details of the clusters that survived non-stationary cluster extent correction are given in Table 1. The extent to which the correlations within each cluster reflect unique variance of STE or SRE was then assessed by checking whether the clusters survived cluster-extent correction thresholding for the equivalent contrasts in the complementary model (i.e. SRE correlation contrasts in the SRE orthogonalised with respect to STE model). These results are reported in the penultimate column (‘Reflects unique variance?’) of Table 1.

**Figure 2:**
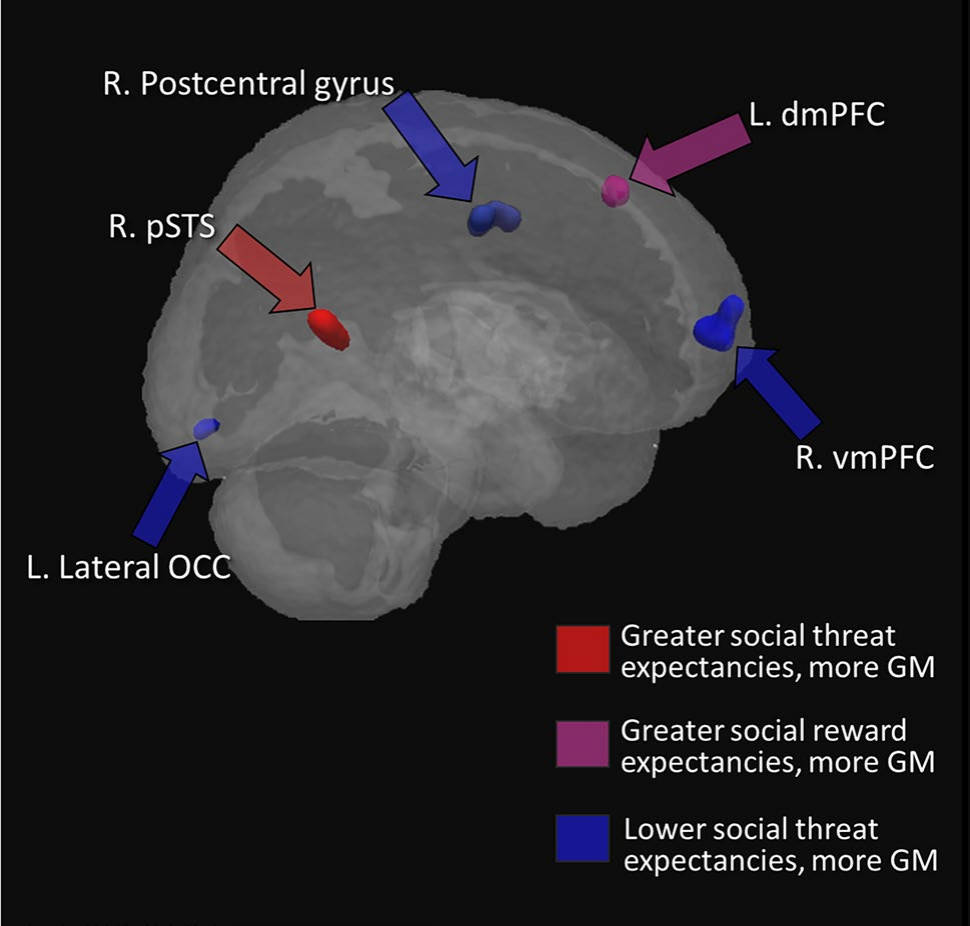
Brain regions in which there were significant associations between self-reported social expectancies and rGMV. For display purposes the clusters are shown at a threshold of *p* < .001, uncorrected.

A positive correlation between SRE and rGMV was found in a dorsomedial region of left prefrontal cortex (dmPFC, see Fig. 2). This result was significant only in the model in which the shared variance was allocated to SRE however; it did not remain significant (at the cluster-size-corrected level) in the model in which the shared variance is allocated to LODESTARS-STE, indicating that this rGMV-expectancy association is partially attributable to shared variance between social reward and threat expectancies. No other correlations (positive or negative) of rGMV with LODESTARS-SRE survived cluster extent correction.

Greater rGMV in right posterior superior temporal sulcus (pSTS) was associated with higher STE (Fig. 2), whereas individuals who reported lower expectancies of social threat had greater GM volumes in right ventromedial PFC (vmPFC, see Fig. 2), left lateral occipital lobe (lOCC, see Fig. 2), and right postcentral gyrus (somatosensory cortex, Fig. 2).

The extent and location of each of the clusters that survived non-stationary extent correction are summarised in Fig. 2.

### Statistical analysis 2: brain regions in which rGMV is correlated with both reward and threat expectancies

**Figure 3:**
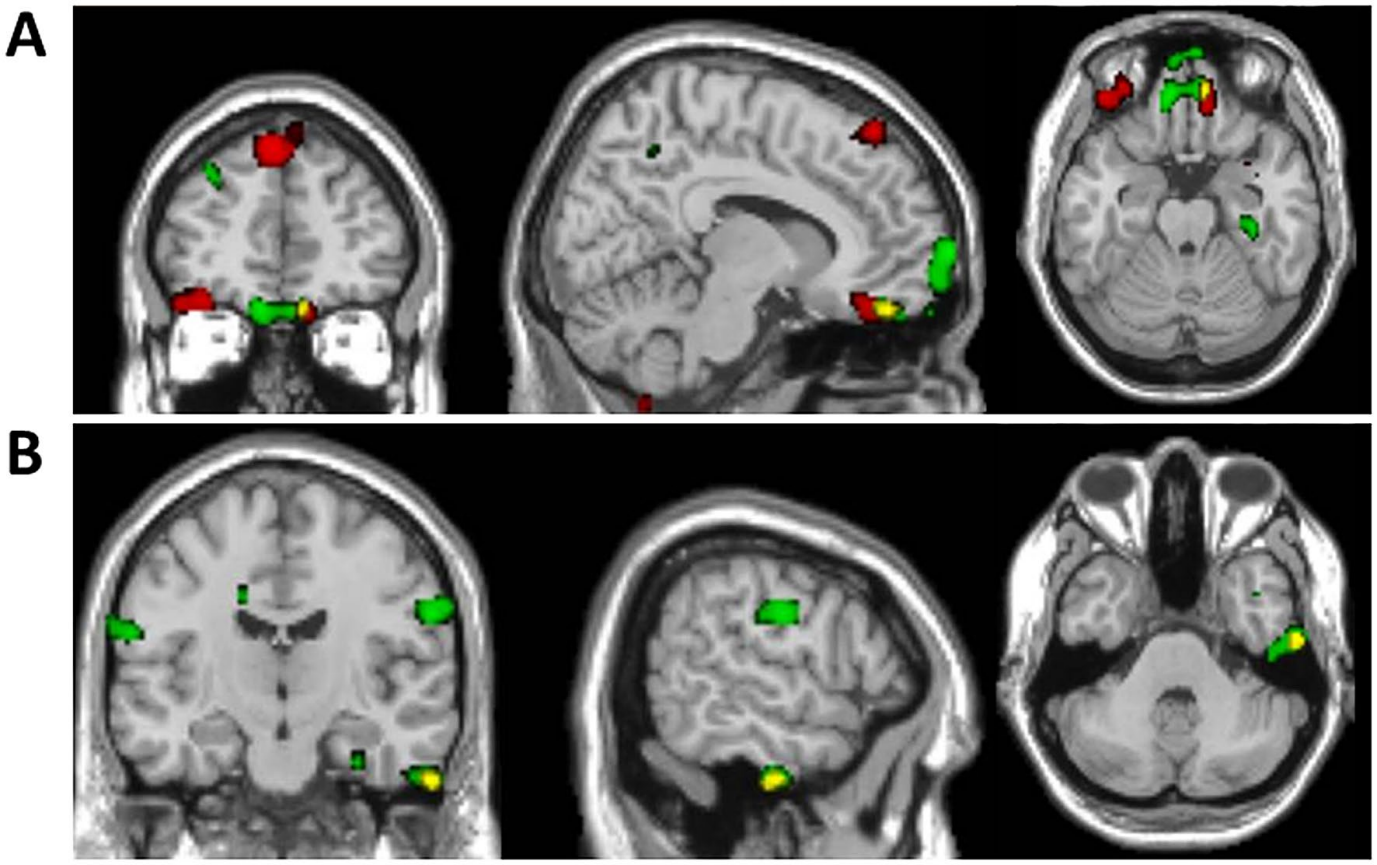
Overlay of regions in which rGMV correlates positively with social reward expectancy and negatively with social threat expectancy. Red = SRE_positive; green = STE_negative; yellow = overlap. The SPMs were thresholded at *p* < 0.005 with 10 voxel minimum cluster extent. (**A**) (upper panel) shows the extent of overlap in right orbitofrontal/ventromedial prefrontal cortex. (**B**) (lower panel) shows the overlap in right lateral inferior temporal gyrus.

The results of these overlap analyses are given in Table 2 and Fig. 3. The only pairing for which there were overlapping clusters (at *p* < 0.005, with 86 voxel extent threshold) was {SRE-positive and STE-negative} (‘bipolar valence’). There was overlap between clusters in the vmPFC (Fig. 3A), in the right lateral inferior temporal gyrus (Fig. 3B) and in right parahippocampal gyrus.

### Statistical analysis 3: Structural covariance analyses

**Figure 4:**
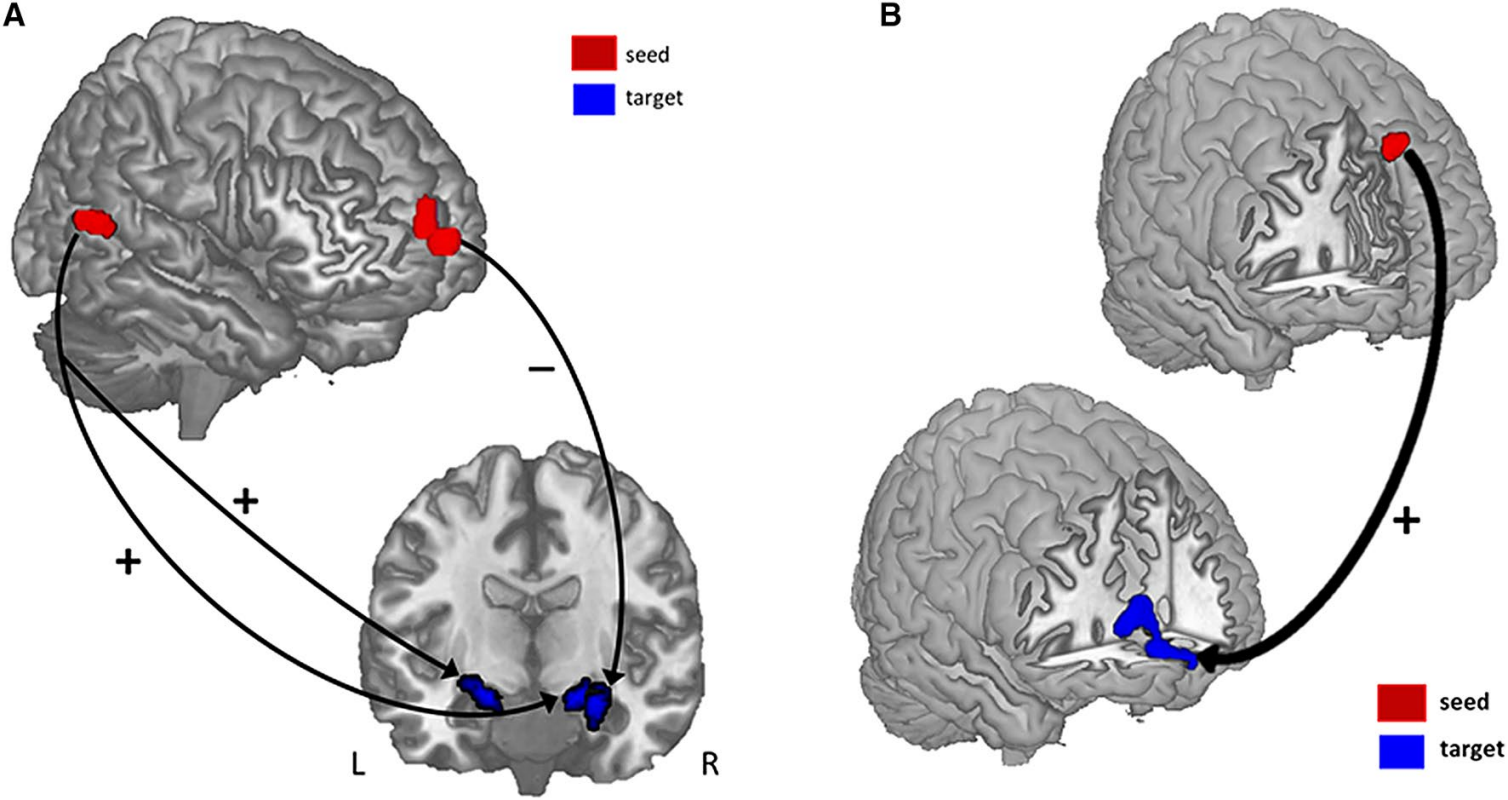
Structural covariance results. (**A**) rGMV in the vmPFC and pSTS seed regions covaried with rGMV in the amygdala. vmPFC and amygdala rGMV were *negatively* correlated, while pSTS and amygdala rGMV were *positively* correlated. (**B**) rGMV in the dmPFC seed region covaried *positively* with rGMV in the vmPFC.

Seed-based SC revealed that pSTS rGMV covaried *positively* with right and left amygdala rGMV (16.5, − 4.5, − 15 and − 22.5, − 12, − 0.5, respectively), while vmPFC rGMV covaried *negatively* with right amygdala rGMV (27, − 6, − 15; see Fig. 4A). rGMV in the dmPFC seed covaried *positively* with rGMV in vmPFC (3, 63, − 7.5; see Fig. 4B).

## Discussion

We report a set of focal brain regions in which regional grey matter volume (rGMV) is associated with individual differences in dispositional expectancies of social reward or threat. The results extend previous functional studies revealing that individual differences in future-oriented emotions are underpinned by a network centred on vmPFC^62^, and additionally point to involvement of other key structures including dmPFC, pSTS and SRC, that have been argued to be important ‘hub’ or ‘nexus’ regions within networks supporting social cognition and interoception, respectively. Further, seed-based structural covariance analyses, showing that vmPFC and pSTS volumes covaried (negatively vs. positively) with those of the amygdala, suggest that these regions may functionally interact with broader networks anchored in the amygdala thought to support unique dispositions for fostering and maintaining social relationships^63^.

Our novel scenario-based measure generated considerable individual differences in both reward and threat expectancies for the imagined social event. Social reward expectancies (SRE) were significantly higher than social threat expectancies (STE). This finding is robust (n > 1,300^40^) and in line with previous research showing that healthy young adults typically anticipate social acceptance and positive social evaluation from novel interpersonal interactions (e.g.^36,64,65^) as part of a more general optimistic view of their personal future^30^. This optimism bias is considered to be adaptive^33^, beneficial for physical health and vital for mental health^30^. SRE were largely independent of STE, although the two were modestly inversely correlated (see also^38,39,66^).

Several prominent models posit that two neurobehavioral systems underlie individual differences in affect and motivation^7,9^. Prospection is at the heart of these models. The appetitive (or approach) system underlies reward pursuit, in part by generating anticipatory and/or anticipated positive emotions. The aversive system underlies anxiety, vigilance, and withdrawal (behavioural inhibition) at the prospect of threat. Our findings align with these models and are broadly consistent with other research showing that social approach and avoidance motives—characterized as the ‘hope for affiliation’ and ‘fear of rejection’ respectively—are distinct dispositions^10,67^. Further, our work and others’ indicates that positive and negative future-related cognitions are best conceived as separate dimensions of experience, differentially associated with anhedonia and anxiety, respectively^30^.

### VBM findings—correlations with social reward expectancies

Previous research shows that the anticipated pleasure from imagined social interactions correlates with the vividness of imagined people and places in such scenes^68^, and complementary, that the spatio-temporal clarity of imagined events is greater for events evoking anticipated positive versus negative affect^69^. Further, dispositional optimism is associated with the tendency to vividly imagine positive events in one's future (e.g.^70^.); whereas anhedonia is associated with reduced capacity to simulate detailed future positive events (e.g.^71,72^.) as well as reduced accessibility of such images^30^. Positive episodic expectancy (‘anticipatory savouring’) in summary requires a vivid, contextually detailed mental representation of future reward.

Here, overlap analysis revealed that rGMVs in vmPFC, parahippocampal cortex (PHC) and ventral anterior temporal lobe (vATL) were positively correlated with social reward expectancies *and* negatively correlated with social threat expectancies. These are all regions of the core remembering-imagining network underpinning MTT (e.g.^32^). Consistent with our VBM results, these regions are more activated during the simulation of positive versus negative events^73^. As part of this network, vmPFC tracks the anticipated positive affective quality of future scenarios^62,74–76^, consistent with a broader role in subjective valuation^77^.

vmPFC tracks subjective value of imagined events as a function of one’s needs^78^ and chronic goals^79^ and is sensitive to individuals’ optimism bias in their *expectancies* about the hedonic rewards (or other benefits) that the participant hopes to obtain from such events^79^. For example, the level of vmPFC activity when imagining positive vs. negative future scenarios is positively correlated with trait optimism^76^. Of particular relevance to our findings, vmPFC activity to anticipated *social* feedback is enhanced when participants have positive expectancies about social outcomes^37,80,81^ (see also^73^).

Functionally, vmPFC interacts with PHC and vATL to produce structured positively-valenced mental representations replete with detailed spatiotemporal context and rich (personal) semantic and sensory details^62^. Our findings that rGMV not only in vmPFC, but also PHC and ATL, is higher in individuals with higher social reward expectancies is congruent with the behavioural work cited above, linking the vividness of future simulations to their reward value, and further relates to the finding of reduced engagement of these regions during prospection in patients with depression and anhedonia^82^.

Positively biased simulations are partly grounded in biased encoding, consolidation and/or retrieval of autobiographical memories^32,83^. Speer et al.^84^ found increased dmPFC activity linked to recall of positive autobiographical memories (‘savouring’). Our finding of greater dmPFC rGMV in people with more positive expectancies further corroborates the neural entwining of autobiographical expectancies and memories^32^. Our finding of positive structural covariance between dmPFC and vmPFC—which potentially reflects long term increased functional connectivity^43,60^—may be because the social context inherent in positive mental constructions enhances their value^62,68^. It is also possible that the reward value of a simulated event may motivate the degree to which participants engage in mentalizing processes subserved by dmPFC^62^.

rGMV in vmPFC, PHC, and vATL were also correlated with lower STE. Reduced vividness of positive future thinking is characteristic of anxious as well as depressed individuals, in addition to anxious expectancies about future social interactions unique to anxiety^72^. Social anxiety can be regarded as a position along a continuum ranging from a lack of anxiety, to mild shyness and then social anxiety disorder (SAD)^15,16,85^, so our findings can meaningfully be compared with studies of SAD, which show reduced vmPFC volume^86,87^.

The correlation of vmPFC rGMV with lower STE and greater SRE concurs with the well-established role of vmPFC in emotion regulation. A large-scale neuroimaging meta-analysis of affect regulation across 3 distinct domains (fear extinction, placebo effects, cognitive reappraisal) identified vmPFC activation as the only ‘common neural regulator’ dampening current and anticipated negative affect^27^.

These results support the hypothesis that vmPFC plays a ubiquitous role in dampening current and anticipated negative affect^27^. Our data extend previous work by indicating that the minimisation of STE—and/or the maintenance low threat expectancies—may be implemented in the brain by similar means as the reduction of fear or negative affect in other emotion regulation scenarios, possibly by self-generating positive emotion in negative situations^88^.

In healthy adults, successful down-regulation of negative affect is consistently associated not only with increased BOLD activity in the vmPFC, but also with concordant reduction of activity in the amygdala^27^. Further, vmPFC damage engenders disinhibited activity of the amygdala and, consequently, elevated levels of negative affect^89^. Our structural covariance findings add further convergent evidence of the regulatory link between these regions by demonstrating a negative correlation between amygdala rGMV and vmPFC rGMV. This negative coupling may reflect the finding that during typical development, amygdala-vmPFC functional connectivity becomes more strongly negative, and in adults, such negative coupling is linked to both reduced amygdala reactivity to social threat and lower anxiety^90^.

### VBM findings—correlations with social threat expectancies

There were several unique rGMV correlates of individual differences in STE. Heightened threat expectancies (fears of potential embarrassment and social rejection) were associated with increased rGMV in right pSTS, alongside decreased rGMV in somatosensory-related cortex (SRC) and lateral occipital cortex (OCC).

Cognitive theories posit that heightened social anxiety results from biased information processing^91^. Alongside regulatory deficits, a processing style marked by hypervigilance and an attentional bias to the social environment for signals of social evaluation is considered a causal and maintaining factor in social anxiety^91^.

Our results are in line with studies suggesting that pSTS serves as an interface between perception of social information and social cognition^92^. pSTS plays a role in analysing socially relevant perceptual information (eye gaze, tone of voice, facial and bodily threat signals), evaluating its implications and orienting and sustaining attention accordingly, in line with the individual’s present affective state and social goals^93^. pSTS rGMV is increased in SAD and shyness (e.g.^87,94^), and increased pSTS activity to social perceptual cues (eye gaze etc.) has been consistently demonstrated in individuals who are social inhibited, shy, and socially anxious^95–98^. Further, resting amygdala–pSTS functional connectivity has been linked to biased social attention and perception in social anxiety^92,99^. Collectively, this work suggests that chronic hypervigilance for threat may result from, or result in, increased rGMV in right pSTS. Increased expectancies of threat when anticipating future situations may be fundamentally underpinned by these attentional biases^91,100,101^.

Heightened attention to threat may lead to enhanced encoding, elaboration, consolidation and retrieval of negatively biased memories^100,102^, resulting in an increased tendency to construct negatively biased expectancies^100^. Further, increased internal attention to threat may maintain attention to negatively constructed future simulations in spontaneous thought, leading to heightened subjective expectancies of their occurrence and increased anticipatory worry^100,103^. In turn, this may lead to repercussive effects with increased expectancies further increasing biased attention^101^.

Our findings thus support “combined cognitive bias” models of anxiety^91^ as we show that the neural structures underpinning attentional biases also underpin prospective ones. Other research has found that pSTS activity is related to remembering and imagining socially threatening situations^24^; and is increased during such simulations in individuals with SAD^104^.

Surprisingly, we *did not* find that amygdala volume directly correlates with individuals’ STE, despite its established role in threat processing, including anticipation of social evaluation^105^ and a proposed role in mediating temperamental shyness^23,106^. However, we did find positive structural co-variation of pSTS with amygdala (alongside, as discussed above, negative structural covariance of vmPFC and amygdala) consistent with bidirectional anatomical connectivity between pSTS and amygdala^107^. The reasons for this null result are unclear and may reflect type II (false negative) error. Alternatively, the influence of the amygdala may primarily be modulatory, influencing structural development in connected cortical regions^90^.

We also found reduced rGMV in left lateral OCC, a region that, together with fusiform gyrus, pSTS and amygdala, forms a face perception network^108^. This may link to fMRI work showing increased pSTS activity to face emotion, but decreased OCC activity (alongside poor face identity recognition) in socially inhibited individuals^109,110^.

Somatosensory-related cortex (SRC), in contrast plays a key role in both interoception^111^ and social aversion^63^. Our finding of greater SRC rGMV associated with lower STE thus align closely with findings that individuals with reduced interoceptive sensitivity report significantly greater uncertainty and worry in anticipation of public speaking^112^. Increased uncertainty in social situations may arise not just because of reduced ability to represent/regulate one’s own interoceptive signals, but also because SRC plays a role in automatic affective empathy via simulation of others’ bodily states. Personal distress (a dysfunctional form of empathy linked with maladaptive emotion regulation and social avoidance) has been shown to be linked to lower rGMV in SRC^113^.

Together, the rGMV correlates of STE we find concur with cognitive models of anxiety^91^, which contend that socially anxious persons simultaneously exhibit altered processing of internal (distress) cues and external stimuli potentially indicative of negative social evaluation.

### Limitations

There are some limitations that should be considered when interpreting our results.

Our study was cross-sectional and so cannot determine whether the relationships between rGMV, SRE and STE arise over time through experience-dependent brain plasticity, or alternatively whether individuals with a specific brain structure are predisposed to acquire different expectancies^13^. Most likely, our findings reflect complex brain-body-environment interactions over development^114,115^. In future, longitudinal or training studies could address this.

The cellular basis of rGMV differences identified by VBM is still poorly understood^60^. Any tissue property (e.g. cell density, cell size, myelination) that affects relaxation times, and hence voxel images on T1-weighted MRI, will influence VBM measures.

Finally, the generalizability of our results is unknown. We deliberately chose to study a population of university students, because of the ecological relevance of joining new social groups^1^. Additionally, participants imagined just one scenario. The scenario was designed, however, to be both sufficiently specific to allow episodic simulation whilst sufficiently generic, such that generalized expectancies (e.g. beliefs, schemas) could be tapped. Previous studies (e.g.^66^), reassuringly, suggest a marked degree of consistency across social situations in reward/threat expectancies.

### Conclusions

We found that inter-individual differences in future-oriented thinking in the social domain are reflected in brain macrostructure. In particular, the extent to which individuals hold optimistic vs. pessimistic expectancies for the hedonic outcomes of an imagined social interaction is reflected in rGMV of key brain regions, most notably vmPFC. Our findings concur with the suggestion that vmPFC may integrate various sources of information to conceive the meaning of events for one’s well-being and future prospects^25^. Our results may reflect a neural embedding of such self-related affective valuation, perhaps accounting for the link between vmPFC macrostructure and adaptive social functioning and well-being^28^.

## Data Availability

The LODESTARS is available to download from https://osf.io/hq5sg/. All unthresholded SPMs produced in these analyses are freely available on Neurovault (https://neurovault.org/collections/5897/). Ethical approval conditions do not permit public sharing of raw MRI data as participants have not provided consent for this.
